# Supplementary material for: Real-World Prevalence and Tolerability of Immune-Related Adverse Events in Older Adults with Non-Small Cell Lung Cancer: A Multi-Institutional Retrospective Study
Source: Cancers (Basel). 2024 Jun 6;16(11):2159. doi: 10.3390/cancers16112159 (PMC11172377; doi:10.3390/cancers16112159)
Supplement: Supplementary file 1 [file cancers-16-02159-s001.zip › cancers-3020782-supplementary.pdf]

Supplementary Figure S1

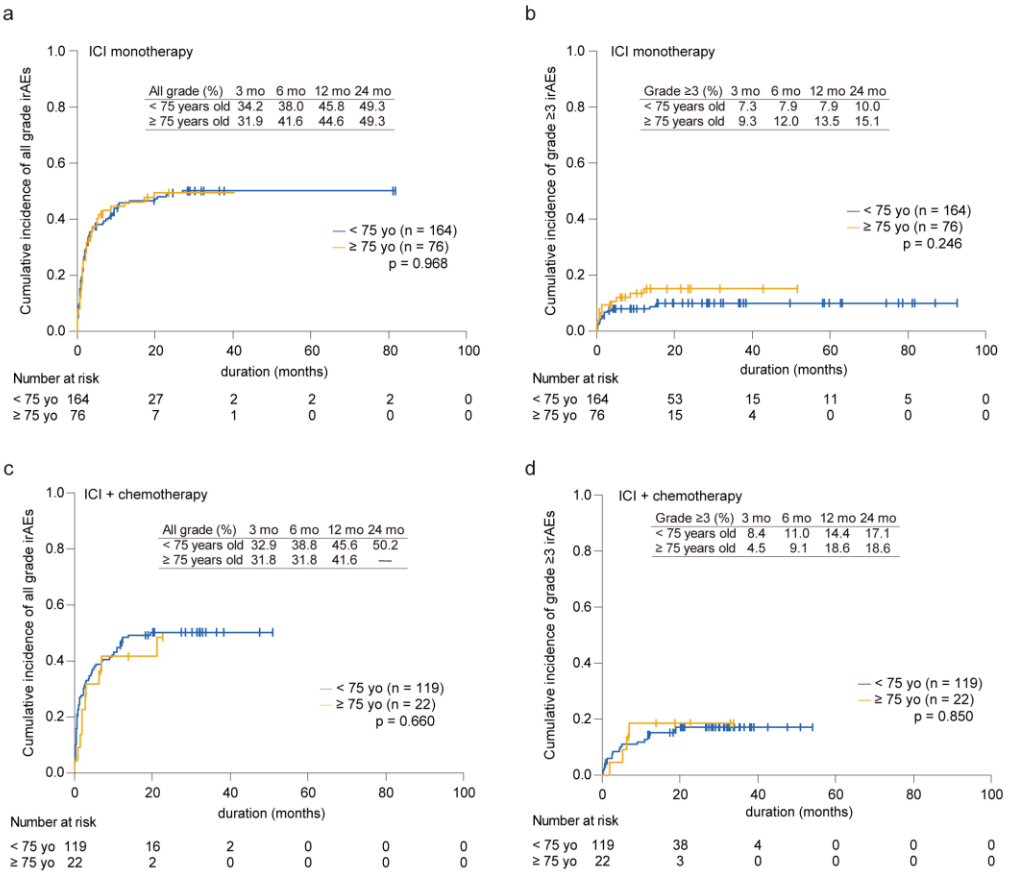

Supplementary Figure S1 Prevalence of immune-related adverse events in ICI monotherapy and ICI combined with chemotherapy

(a) Cumulative incidence of all grade and (b) grade ≥3 immune-related adverse events (irAEs) in younger (< 75 years old, n = 164) and older (≥ 75 years old, n = 76) patients treated with ICI monotherapy. (c) Cumulative incidence of all grade and (d) grade ≥3 immune-related adverse events (irAEs) in younger (< 75 years old, n = 119) and older (≥ 75 years old, n = 22) patients treated with ICI monotherapy.

Significance was determined using the Gray test.

Supplementary Table S1: Patient cohort characteristics after propensity score matching

Characteristic	< 75 years age (n = 103)		≥ 75 years age (n = 103)		*p*
**Age, median—years (range)**	67	(41 - 74)	78	(75 - 89)	**<0.001**
**Sex—no. (%)**					0.999
Male	86	(83.5)	85	(82.5)	
Female	17	(16.5)	18	(17.5)	
**ECOG PS—no. (%)**					0.999
0-1	90	(87.4)	90	(87.4)	
≥ 2	13	(12.6)	13	(12.6)	
**Histology—no. (%)**					0.187
Adenocarcinoma	64	(62.1)	60	(58.3)	
Squamous	24	(23.3)	34	(33.0)	
Other	15	(14.6)	9	(8.7)	
**PD-L1 status—no. (%)**					0.737
TPS ≥ 50%	44	(42.7)	37	(35.9)	
TPS 1-49%	34	(33.0)	36	(35.0)	
TPS < 1%	12	(11.7)	16	(15.5)	
not investigated	13	(12.6)	14	(13.6)	
**Common sites of metastasis—no. (%)**					
brain	28	(27.2)	22	(21.4)	0.417
bone	27	(26.2)	31	(30.1)	0.642
liver	4	(3.9)	7	(6.8)	0.537
**Treatment line—no. (%)**					0.999
1st line	63	(61.2)	62	(60.2)	
2nd line	25	(24.3)	25	(24.3)	
3rd line or more	15	(14.6)	16	(15.5)	
**Administrated ICIs—no. (%)**					0.999
Anti-PD-1/PD-L1 monotherapy	74	(71.8)	75	(72.8)	
- nivolumab	27	(26.2)	20	(19.4)	
- pembrolizumab	46	(44.7)	45	(43.7)	
- atezolizumab	1	(1.0)	10	(9.7)	
Anti-PD-1/PD-L1 with chemotherapy	23	(22.3)	22	(21.4)	
- pembrolizumab + chemotherapy	20	(19.4)	17	(16.5)	
- atezolizumab + chemotherapy	3	(2.9)	5	(4.9)	
Anti-PD-1 + anti-CTLA-4 combination	6	(5.8)	6	(5.8)	
- nivolumab + ipilimumab	3	(2.9)	3	(2.9)	
- nivolumab + ipilimumab + chemotherapy	3	(2.9)	3	(2.9)	

ECOG PS, Eastern Cooperative Oncology Group Performance Status; PD-L1, programmed cell death ligand-1; TPS, tumor proportion score; ICI, immune checkpoint inhibitor; PD-1, programmed cell death protein-1; CTLA-4, cytotoxic T-lymphocyte associated protein-4

Supplementary Figure S2

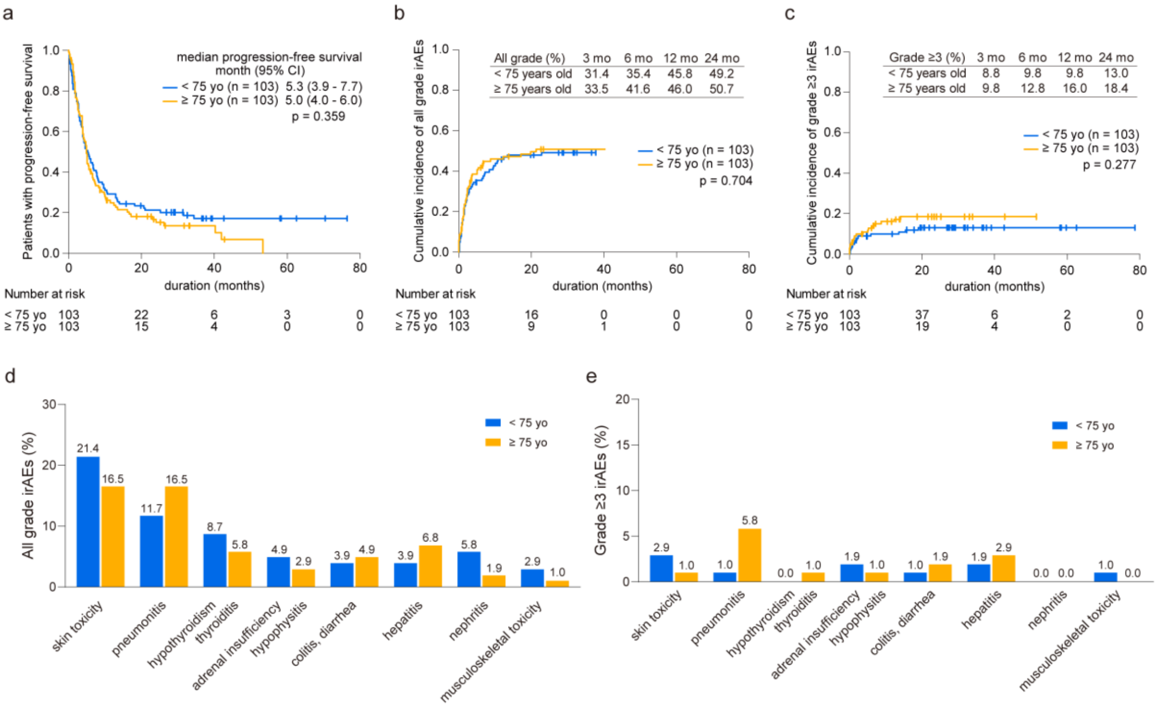

Supplementary Figure S2 Prevalence of immune-related adverse events in propensity score matched younger and older patients

(a) Kaplan–Meier curve analysis for progression-free survival in propensity score matched younger (< 75 years old, n = 103) and older (≥ 75 years old, n = 103) patients. (b) Cumulative incidence of all grade and (c) grade ≥3 immune-related adverse events (irAEs). The prevalence of irAEs is calculated at 3, 6, 12, and 24 months. (d) Comparison of organ-specificity in all grade and (e) grade ≥3 irAEs between younger and older patients.

Significance was determined using the (a) log-rank test, (b, c) Gray test, and (d, e) Fisher’s exact test.

Supplementary Table S2: Association between immune-related adverse events incidence and clinical variable in younger patients

Characteristic	without irAE (n = 152)		with irAE (n = 180)		*p*
**Age, median—years (range)**	66	(39 - 74)	78	(36 - 74)	0.966
**Sex—no. (%)**					
Male	110	(72.4)	150	(83.3)	**0.017**
Female	42	(27.6)	30	(16.7)	
**ECOG PS—no. (%)**					
0-1	126	(82.9)	165	(91.7)	**0.019**
≥ 2	26	(17.1)	15	(8.3)	
**Histology—no. (%)**					
Adenocarcinoma	100	(65.8)	109	(60.6)	0.54
Squamous	37	(24.3)	47	(26.1)	
Other	15	(9.9)	24	(13.3)	
**PD-L1 status—no. (%)**					
TPS ≥ 50%	47	(30.9)	64	(35.6)	0.328
TPS 1-49%	47	(30.9)	54	(30.0)	
TPS < 1%	28	(18.4)	39	(21.7)	
not investigated	30	(19.7)	23	(12.8)	
**Common sites of metastasis—no. (%)**					
brain	43	(28.3)	45	(25.0)	0.534
bone	55	(36.2)	48	(26.7)	0.074
liver	13	(8.6)	19	(10.6)	0.58
**Treatment line—no. (%)**					
1st line	81	(53.3)	117	(65.0)	0.084
2nd line	37	(24.3)	35	(19.4)	
3rd line or more	34	(22.4)	28	(15.6)	
**Administrated ICIs—no. (%)**					
Anti-PD-1/PD-L1 monotherapy	84	(55.3)	80	(44.4)	NA
- nivolumab	38	(25.0)	38	(21.1)	
- pembrolizumab	37	(24.3)	39	(21.7)	
- atezolizumab	9	(5.9)	3	(1.7)	
Anti-PD-1/PD-L1 with chemotherapy	60	(39.5)	59	(32.8)	
- pembrolizumab + chemotherapy	44	(28.9)	49	(27.2)	
- atezolizumab + chemotherapy	16	(10.5)	10	(5.6)	
Anti-PD-1 + anti-CTLA-4 combination	8	(5.3)	41	(22.8)	
- nivolumab + ipilimumab	3	(2.0)	7	(3.9)	
- nivolumab + ipilimumab + chemotherapy	5	(3.3)	34	(18.9)	

ECOG PS, Eastern Cooperative Oncology Group Performance Status; PD-L1, programmed cell death ligand-1; TPS, tumor proportion score; ICI, immune checkpoint inhibitor; PD-1, programmed cell death protein-1; CTLA-4, cytotoxic T-lymphocyte associated protein-4

Supplementary Table S3: Association between immune-related adverse events incidence and clinical variable in older patients

Characteristic	without irAE (n = 54)		with irAE (n = 50)		*p*
**Age, median—years (range)**	78	(75 - 89)	78	(75 - 89)	0.839
**Sex—no. (%)**					
Male	43	(79.6)	43	(86.0)	0.455
Female	11	(20.4)	7	(14.0)	
**ECOG PS—no. (%)**					
0-1	44	(81.5)	47	(94.0)	0.075
≥ 2	10	(18.5)	3	(6.0)	
**Histology—no. (%)**					
Adenocarcinoma	31	(57.4)	30	(60.0)	0.107
Squamous	21	(38.9)	13	(26.0)	
Other	2	(3.7)	7	(14.0)	
**PD-L1 status—no. (%)**					
TPS ≥ 50%	19	(35.2)	18	(36.0)	0.499
TPS 1-49%	18	(33.3)	19	(38.0)	
TPS < 1%	11	(20.4)	5	(10.0)	
not investigated	6	(11.1)	8	(16.0)	
**Common sites of metastasis—no. (%)**					
brain	10	(18.5)	12	(24.0)	0.632
bone	20	(37.0)	12	(24.0)	0.202
liver	4	(7.4)	3	(6.0)	> 0.999
**Treatment line—no. (%)**					
1st line	28	(51.9)	35	(70.0)	0.063
2nd line	18	(33.3)	7	(14.0)	
3rd line or more	8	(14.8)	8	(16.0)	
**Administrated ICIs—no. (%)**					
Anti-PD-1/PD-L1 monotherapy	40	(74.1)	36	(72.0)	0.422
- nivolumab	13	(24.1)	7	(14.0)	
- pembrolizumab	23	(42.6)	23	(46.0)	
- atezolizumab	4	(7.4)	6	(12.0)	
Anti-PD-1/PD-L1 with chemotherapy	12	(22.2)	10	(20.0)	
- pembrolizumab + chemotherapy	9	(16.7)	8	(16.0)	
- atezolizumab + chemotherapy	3	(5.6)	2	(4.0)	
Anti-PD-1 + anti-CTLA-4 combination	2	(3.7)	4	(8.0)	
- nivolumab + ipilimumab	2	(2.0)	1	(2.0)	
- nivolumab + ipilimumab + chemotherapy	0	(0.0)	3	(6.0)	

ECOG PS, Eastern Cooperative Oncology Group Performance Status; PD-L1, programmed cell death ligand-1; TPS, tumor proportion score; ICI, immune checkpoint inhibitor; PD-1, programmed cell death protein-1; CTLA-4, cytotoxic T-lymphocyte associated protein-4
